# Itaconic Acid Alleviates Perfluorooctanoic Acid-Induced Oxidative Stress and Intestinal Damage by Regulating the Keap1/Nrf2/Ho-1 Pathway and Reshaping the Gut Microbiota

**DOI:** 10.3390/ijms25189826

**Published:** 2024-09-11

**Authors:** Lianchi Wu, Zhaoying Hu, Xinyu Luo, Chaoyue Ge, Yujie Lv, Shenao Zhan, Weichen Huang, Xinyu Shen, Dongyou Yu, Bing Liu

**Affiliations:** 1 College of Animal Sciences, Zhejiang University, Hangzhou 310058, China; 2 ZJU-Xinchang Joint Innovation Centre (TianMu Laboratory), Gaochuang Hi-Tech Park, Shaoxing 312500, China

**Keywords:** itaconic acid, perfluorooctanoic acid, oxidative stress, inflammation

## Abstract

Itaconic acid (IA) is recognized for its potential application in treating intestinal diseases owing to the anti-inflammatory and antioxidant properties. Perfluorooctanoic acid (PFOA) can accumulate in animals and result in oxidative and inflammatory damages to multi-tissue and organ, particularly in the intestinal tract. This study aimed to explore whether IA could mitigate intestinal damage induced by PFOA exposure in laying hens and elucidate its potential underlying mechanisms. The results showed that IA improved the antioxidant capacity of laying hens and alleviated the oxidative damage induced by PFOA, as evidenced by the elevated activities of T-SOD, GSH-Px, and CAT, and the decreased MDA content in both the jejunum and serum. Furthermore, IA improved the intestinal morphological and structural integrity, notably attenuating PFOA-induced villus shedding, length reduction, and microvillus thinning. IA also upregulated the mRNA expression of *ZO-1*, *Occludin*, *Claudin-1*, and *Mucin-2* in the jejunum, thereby restoring intestinal barrier function. Compared with the PF group, IA supplementation downregulated the gene expression of *Keap1* and upregulated the *HO-1*, *NQO1*, *SOD1*, and *GPX1* expression in the jejunum. Meanwhile, the PF + IA group exhibited lower expressions of inflammation-related genes (*NF-κB*, *IL-1β*, *IFN-γ*, *TNF-α*, and *IL-6*) compared to the PF group. Moreover, IA reversed the PFOA-induced imbalance in gut microbiota by reducing the harmful bacteria such as *Escherichia-Shigella*, *Clostridium innocuum*, and *Ruminococcus torques*, while increasing the abundance of beneficial bacteria like *Lactobacillus*. Correlation analysis further revealed a significant association between gut microbes, inflammatory factors, and the Keap1/Nrf2/HO-1 pathway expression. In conclusion, dietary IA supplementation could alleviate the oxidative and inflammatory damage caused by PFOA exposure in the intestinal tract by reshaping the intestinal microbiota, modulating the Keap1/Nrf2/HO-1 pathway and reducing oxidative stress and inflammatory response, thereby promoting intestinal homeostasis.

## 1. Introduction

Per- and polyfluoroalkyl substances (PFAS) consist of thousands of synthetic chemicals that have been extensively utilized in industrial production for decades [1]. Perfluorooctanoic acid (PFOA) is the most common type of PFAS. Owing to its superior thermal stability and hydrophobic and oleophobic properties, PFOA is used not only in industrial fields such as chemical plating but also in consumer products such as food packaging [2]. However, its high stability makes it prone to significant residues in animals, plants, and the environment. PFOA residues have been detected in both commercially captive and backyard free-range laying hens, possibly originating from contaminated soil and feed [3]. More seriously, the deposition of PFOA in the body has varying degrees of toxic effects on humans and animals, inevitably leading to damage to various tissues and organs [4]. Therefore, it is important to study the mechanism of PFOA toxicity in laying hens and explore effective ways to reduce its toxicity.

There is growing evidence that oxidative damage following exposure to PFOA is a key mechanism of PFAS toxicity [5]. PFOA affects the total antioxidant capacity and enzyme activities of the organism through the Keap1/Nrf2 pathway [6,7], disrupts the homeostasis of the antioxidant system [8], prevents the antioxidant system from activating its defense mechanisms [9], induces excessive production and accumulation of reactive oxygen species [10], and ultimately causes damage to the organism [11]. Furthermore, exposure to PFOA-induced oxidative stress exacerbates the immune inflammatory response. PFOA exposure has been reported to increase lipid peroxidation and impair the cholinergic system, thereby aggravating inflammation in the gut [12]. Additionally, PFOA has been strongly associated with colitis and plays a significant role in exacerbating the condition [13,14].

Intestinal homeostasis is crucial for maintaining organismal health, defending against pathogens, and preventing diseases. This relies on maintaining epithelial cell integrity through gut microbiota, modulating the intestinal mucosal immune system, and their interactions [15,16]. Increasing evidence indicates that exposure to harmful chemicals, such as PFAS, alters gut microbiota composition, thereby impacting host health. PFOA has been reported to induce intestinal metabolic disorders in mice by altering the gut microbiota [17]. Therefore, investigating the effects of PFOA on the intestinal microbiota is crucial for reducing oxidative stress and alleviating intestinal damage in laying hens.

The Keap1/Nrf2 signaling pathway is a major defense mechanism against oxidative stress in vivo and plays a crucial role in various oxidative stress-related diseases, including cancer, neurodegenerative disorders, and non-alcoholic fatty liver disease [18,19,20]. Itaconic acid (IA) is a by-product of the tricarboxylic acid cycle, produced by decarboxylation via cis-aconitine decarboxylase. As a novel small molecule metabolite and renewable organic acid, it has garnered extensive attention from researchers in recent years and is now listed among the top twelve value-added products [21]. IA exerts significant protective effects in oxidative stress and inflammatory responses by activating Keap1/Nrf2 pathway, which regulates the expression of downstream antioxidant and anti-inflammatory genes [22,23]. Therefore, investigating the activation of the Keap1/Nrf2 signaling pathway by itaconic acid is vital for the prevention and treatment of oxidative stress-induced diseases. Additionally, IA exhibits broad antimicrobial effects that protect the organism against pathogenic bacterial invasion. In a Klebsiella pneumoniae infection model, IA effectively attenuated pernicious bacteria-induced intestinal homeostatic imbalance by optimizing the gut microbiota [24]. However, whether IA can regulate the Keap1/Nrf2 signaling pathway and alleviate oxidative damage of perfluorinated compounds through intestinal flora has not been re-ported.

This study aimed to investigate whether IA can alleviate intestinal damage caused by PFOA exposure in hens and to elucidate its underlying mechanisms. We analyzed the damage caused by PFOA to the intestinal tract of laying hens by assessing their oxidative stress status and intestinal barrier function. Furthermore, we revealed the protective effects of IA by analyzing changes in intestinal microbial composition and oxidative and inflammation-related pathways. Lastly, we explored the correlation between oxidative stress, inflammation, and intestinal microbes to provide a reference for improving intestinal homeostasis in laying hens.

## 2. Results

### 2.1. Serum and Jejunum Antioxidant Capacity

**Figure 1:**
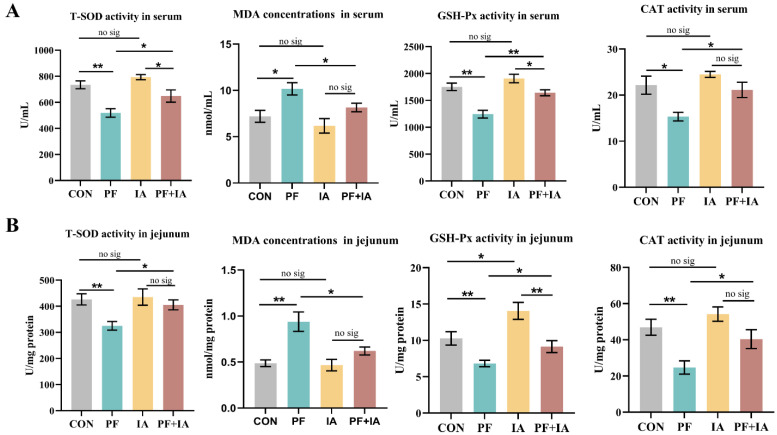
The changes in the antioxidant capacity of serum and jejunum. (**A**) Serum antioxidant capacity; (**B**) Antioxidant capacity of the jejunum. Results are means ± SEM; *n* = 6; * *p* < 0.05, ** *p* < 0.01.

As shown in Figure 1, PFOA exposure significantly decreased the activities of T-SOD, GSH-Px, and CAT (*p* < 0.05 or *p* < 0.01) and significantly increased the MDA content (*p* < 0.05) in serum compared to the CON group. In addition, the activities of T-SOD, GSH-Px, and CAT were significantly reduced (*p* < 0.01) in the jejunum, and MDA content (*p* < 0.01) was higher in the PF group. However, the PF + IA group significantly increased the activities of T-SOD, GSH-Px, and CAT (*p* < 0.05 or *p* < 0.01) and decreased the MDA content (*p* < 0.05) in serum compared to the PF group. Comparable findings were observed in the jejunum, where IA treatment significantly increased the activities of T-SOD, GSH-Px, and CAT (*p* < 0.05) and significantly decreased the MDA content (*p* < 0.05).

### 2.2. Jejunum Morphology

**Figure 2:**
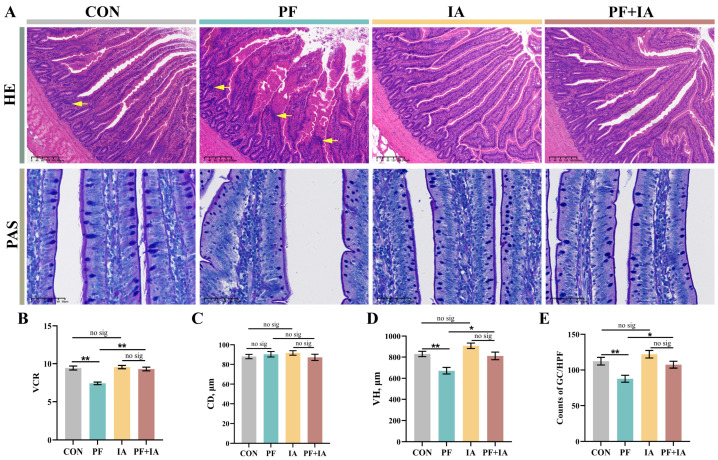
The changes in the morphology of the jejunum in laying hens. (**A**) Photograph of representative jejunum HE and PAS staining. Quantification of VH (**B**), CD (**C**), VCR (**D**), and counts of GC (**E**). GC, goblet cells; CD, crypt depth; VH, villus height; VCR, the ratio of VH to CD. The Yellow arrows indicate inflammatory cell infiltration. Results are means ± SEM; *n* = 6; * *p* < 0.05, ** *p* < 0.01.

The HE staining are shown in Figure 2A. Compared to the CON group, the jejunal structure in the PF group showed significant damage, as evidenced by the shedding of villous epithelial cells, broken villi, and poorly defined borders with infiltration of immune cells in lamina propria. However, this was alleviated by treatment with IA. The intestinal morphology of the PF + IA group was restored to normal, with well-aligned villi. Additionally, VH and VCR were significantly lower (*p* < 0.01) in the PF group compared to the CON group, but significantly higher after IA treatment (Figure 2B–D). Similar results were observed with AB-PAS staining. The count of goblet cells was lower in the PF group compared to the CON group (*p* < 0.01), but significantly improved (*p* < 0.05) in the PF + IA group (Figure 2E).

### 2.3. Intestinal Barrier Function

**Figure 3:**
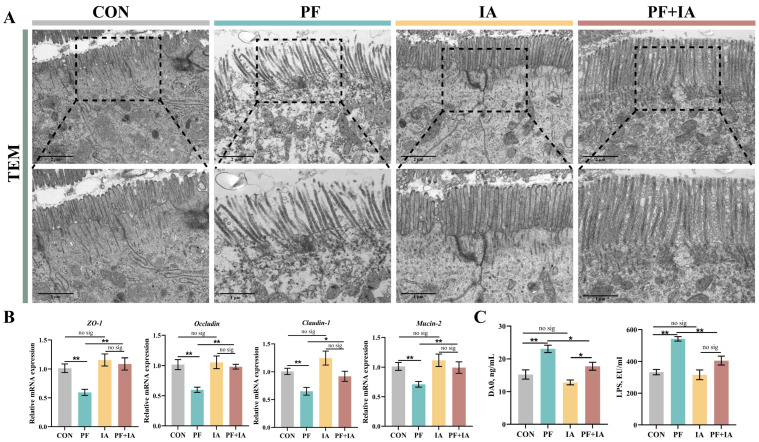
The changes in intestinal barrier function of the jejunum. (**A**) TEM images of the jejunum. (**B**) Relative mRNA levels of tight junction proteins. (**C**) Serum indicators of intestinal permeability. Results are means ± SEM; *n* = 6; * *p* < 0.05, ** *p* < 0.01.

**Figure 4:**
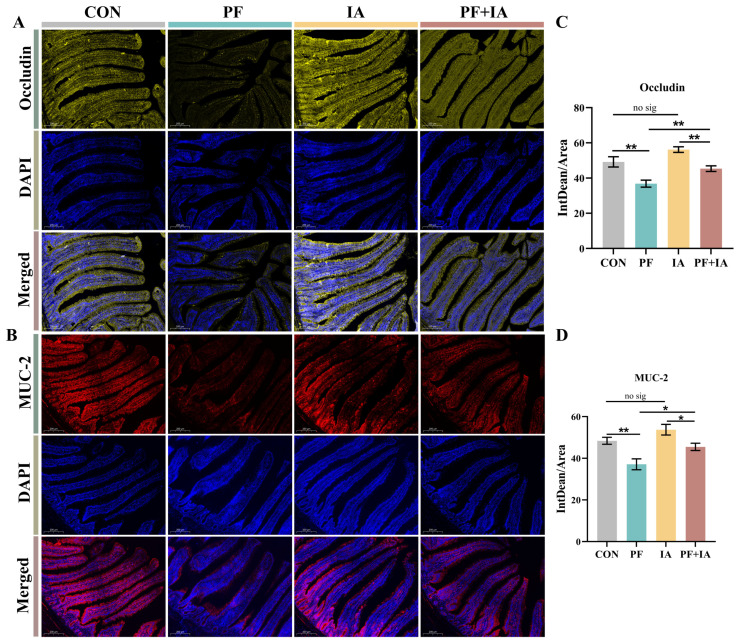
The changes in the expression of jejunal tight junction proteins. (**A**) Representative immunofluorescence photomicrographs for Occludin. (**B**) Fluorescence intensity analysis of Occludin. (**C**) Representative immunofluorescence photomicrographs for MUC-2. (**D**) Fluorescence intensity analysis of MUC-2. Results are means ± SEM; *n* = 6; * *p* < 0.05, ** *p* < 0.01.

The TEM results demonstrated that the microvilli in the CON group were neatly arranged, with complete tight junction structures (Figure 3A). By contrast, the villi in the PF group were sparsely arranged, with large gaps and breaks, and unclear tight junctions. However, both the IA and PF + IA groups exhibited complete microvilli and normal tight junction structures. In addition, serum indicators of intestinal permeability, DAO and LPS, were significantly higher in the PF group compared to the CON group, but were significantly lower after IA treatment (Figure 3C). Similarly, the assessment of the key tight junction proteins expression showed consistent results (Figure 3B). The expression of *Occludin*, *Mucin-2*, *Claudin-1*, and *ZO-1* were lower (*p* < 0.01) in the PF group compared to the CON group but were upregulated (*p* < 0.05) after IA treatment. Consistent with this, immunofluorescence results for Occludin and Mucin-2 also showed lower expression in the PF group compared to the CON group, but the fluorescence intensity increased in the PF + IA group after IA intervention (Figure 4). 

### 2.4. Expression of Keap1/Nrf2/HO-1 Pathway-Related Genes in Jejunum

**Figure 5:**
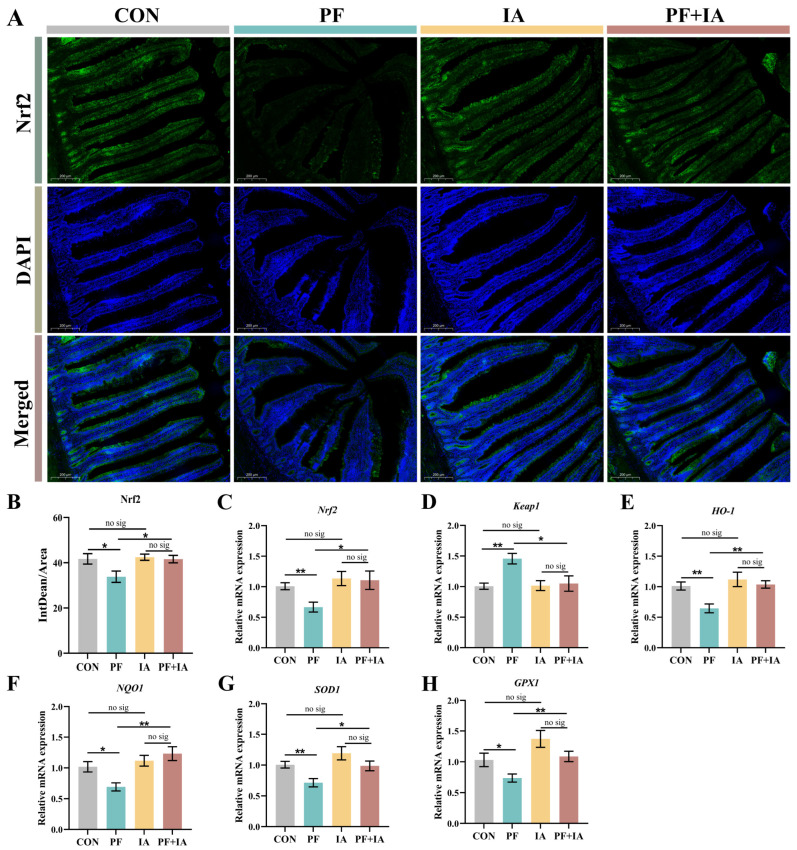
The changes in the jejunal Keap1/Nrf2/HO-1 pathway. (**A**) Representative immunofluorescence photomicrographs for Nrf2. (**B**) Fluorescence intensity analysis of Nrf2. The expression of (**C**) Nrf2, (**D**) Keap1, (**E**) HO-1, (**F**) NQO1, (**G**) SOD1, and (**H**) GPX1 mRNA. Results are means ± SEM; *n* = 6; * *p* < 0.05, ** *p* < 0.01.

Immunofluorescence results showed lower expression of Nrf2 in the PF group compared to the CON group, which increased following IA treatment (Figure 5A,B). Similarly, the expression of mRNA for *Nrf2* was upregulated (*p* < 0.05) in the PF + IA group compared to the PF group (Figure 5C). Additionally, the PF group significantly upregulated the expression of *Keap1* (*p* < 0.01) and significantly downregulated the expression of *HO-1*, *NQO1*, *SOD1*, and *GPX1* (*p* < 0.05 or *p* < 0.05) compared to the CON group (Figure 5D–H). However, this was reversed after IA intervention. The expression level of *Keap1* was decreased (*p* < 0.05) in the PF + IA group compared to the PF group, while the expression levels of *HO-1*, *NQO1*, *SOD1*, and *GPX1* (*p* < 0.05 or *p* < 0.05) were significantly increased.

### 2.5. Gene Expression of Inflammatory Factors in Jejunum

**Figure 6:**
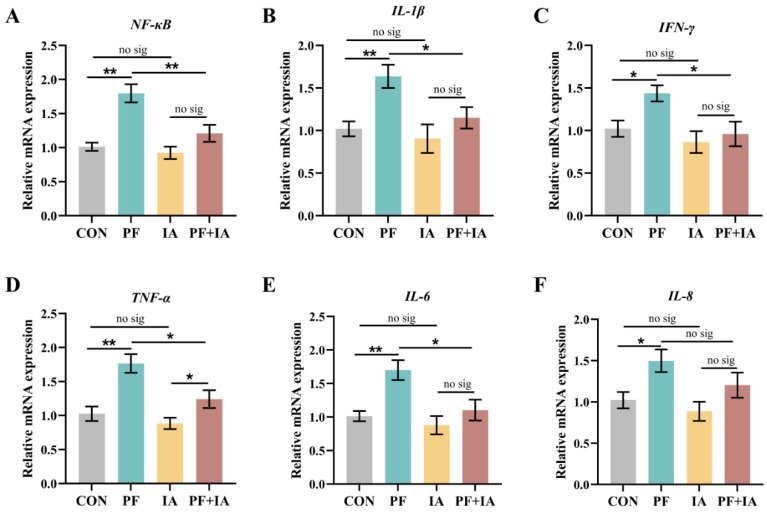
The changes in gene expression of jejunal inflammatory factors. The expression of (**A**) NF-κB, (**B**) IL-1β, (**C**) IFN-γ, (**D**) TNF-α, (**E**) IL-6, and (**F**) IL-8 mRNA. Results are means ± SEM; *n* = 6; * *p* < 0.05, ** *p* < 0.01.

The expression levels of *NF-κB*, *IL-1β*, *IFN-γ*, *TNF-α*, *IL-6*, and *IL-8* were significantly upregulated (*p* < 0.05 or *p* < 0.01) in the PF group compared to the CON group (Figure 6). However, IA treatment reduced the expression of these pro-inflammatory factors. Specifically, the expression of *NF-κB*, *IL-1β*, *IFN-γ*, *TNF-α*, and *IL-6* were downregulated (*p* < 0.05 or *p* < 0.01) in the PF + IA group compared to the PF group.

### 2.6. Microbial Structure and Community

**Figure 7:**
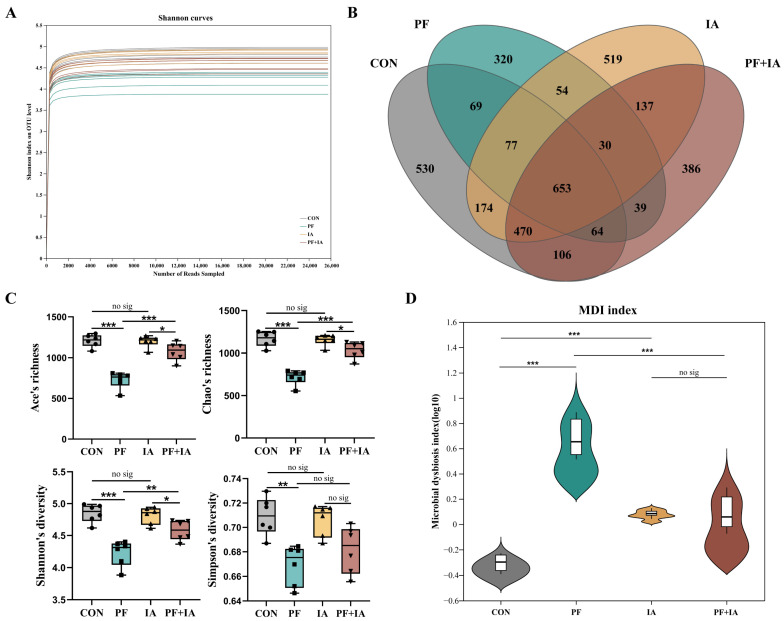
The changes in the composition of the cecal microbiota. (**A**) Rarefaction curves. (**B**) OUT Venn. (**C**) α diversity indices. (**D**) microbial dysbiosis index. * *p* < 0.05, ** *p* < 0.01, *** *p* < 0.001.

The species accumulation curve plateaued, suggesting sufficient sampling for advancing to subsequent analytical stages (Figure 7A). Figure 7B shows the CON, PF, IA, and PF + IA groups contained 530, 320, 519, and 386 unique OTUs, respectively. Ace, Chao, Simpson, and Shannon indices were reduced in the PF group compared to the CON group but reversed after IA treatment, suggesting that IA could elevate α diversity induced by PFOA exposure (Figure 7C). In addition, as shown in Figure 7D, the degree of microbial dysbiosis was greater in PF group but was restored after IA treatment.

**Figure 8:**
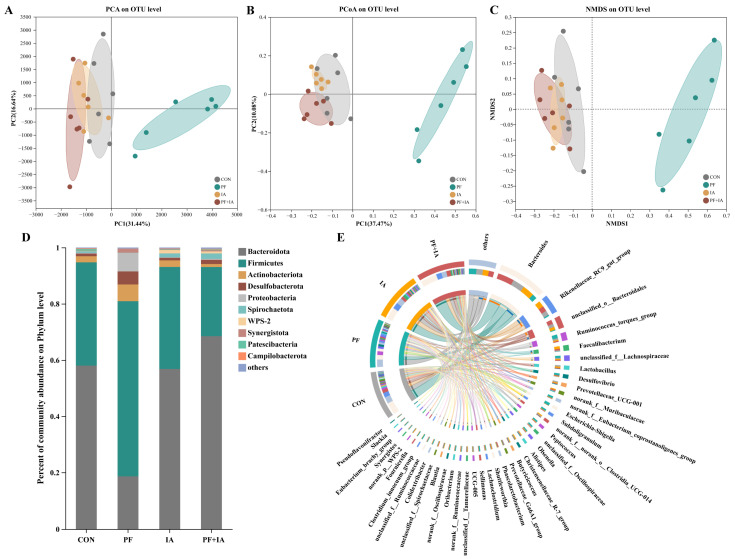
The alterations in the composition of the cecal microbiota. (**A**) Comparison of β diversity indices. (**A**) Principal Component Analysis (PCA). (**B**) Principal co-ordinates analysis (PCoA). (**C**) Non-metric multidimensional scaling analysis (NMDS). (**D**) Microbiota composition at the phylum level. (**E**) Microbiota composition at the genus level.

PCA, PCoA, and NMDS analyses showed significant separation between the PF group and the other groups, with significant differences in β-diversity distances (Figure 8A–C), suggesting that significant changes in colony structure caused by PFOA were corrected as results of IA treatment. Figure 8D shows the abundance between groups at phylum level, it is noteworthy that Proteobacteria were significantly enriched in the PF group. The circus diagram (Figure 8E) shows compositional differences between groups at genus level, indicating significant differences in dominant genera among treatment groups. 

**Figure 9:**
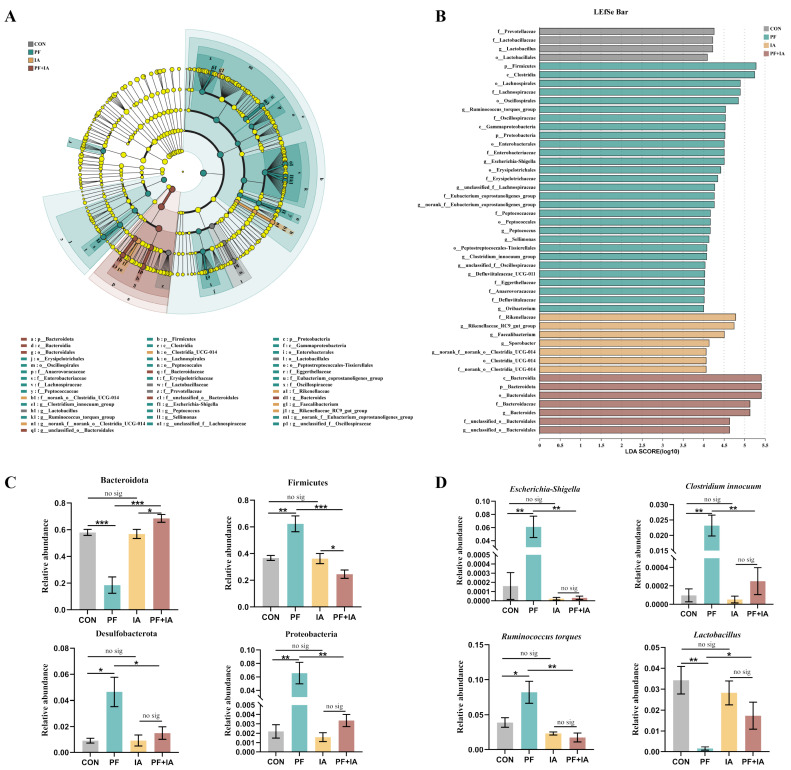
The changes in the cecal microbiota composition. (**A**) The cladogram of LEfSe analysis. (**B**) The histogram of LEfSe analysis. (**C**) Differences in microbiota composition at phylum level. (**D**) Differences in microbiota composition at genus level. * *p* < 0.05, ** *p* < 0.01, *** *p* < 0.001.

Microbiota with significant differences were analyzed using LEfSe (LDA score > 4), revealing 47 distinctive taxa between the four groups (Figure 9A,B). Further determination of the abundance of different bacteria among the groups showed that the bacterial phyla with higher abundance (Firmicutes, Desulfobacterota, and Proteobacteria) in PF group compared to CON group were all modulated by IA treatment (Figure 9C). At the genus level, *Escherichia-Shigella*, *Clostridium innocuum*, and *Ruminococcus torques*, which were enriched in the PF group, were significantly reduced, while the abundance of *Lactobacillus* was increased after IA treatment (Figure 9D).

### 2.7. Correlation Analysis

**Figure 10:**
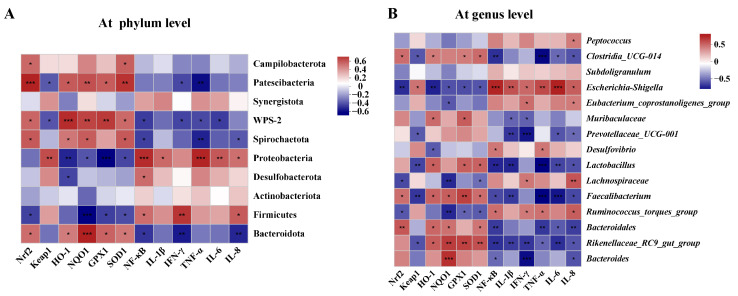
Correlation analysis between gut microbiota and oxidative stress and inflammatory parameters. (**A**) At family level. (**B**) At genus level. * *p* < 0.05, ** *p* < 0.01, *** *p* < 0.001.

Spearman correlation analyses were conducted to investigate associations between gut microbiota and parameters related to oxidative stress and inflammation (Figure 10). Correlation analysis revealed significant positive correlations between Proteobacteria at the phylum level and the expression of *Keap1* and inflammatory factors (*IL-1β*, *NF-κB*, *TNF-α*, *IL-6*, and *IL-8*). Additionally, Proteobacteria showed significant negative correlations with antioxidant genes downstream of the Keap1/Nrf2/HO-1 pathway (*HO-1*, *NQO1*, *SOD1* and *GPX1*). At the genus level, both *Escherichia-Shigella* and *Ruminococcus torques* were significantly negatively correlated with Keap1/Nrf2/HO-1 pathway-related genes (*Nrf2*, *NQO1*, *SOD1* and *GPX1*) and significantly positively correlated with inflammatory factors (*NF-κB*, *TNF-α*, *IL-8* and *IFN-γ*). 

## 3. Discussion

PFOA is a chemical contaminant widely present in the environment and accumulated in living organisms. Its adverse health effects have been demonstrated in both animal and cellular tests [25,26]. Previous studies have found that PFOA disrupts the structural integrity of the intestinal tract and affects intestinal function, leading to intestinal damage in mice [27]. But its potential impact on the health of poultry, particularly the intestinal tract, remains unclear. Recently, IA has been considered for potential application in treating intestinal diseases due to its significant anti-inflammatory, antioxidant, and antibacterial effects [28,29]. However, the protective role and underlying mechanisms of IA against PFOA-induced intestinal damage in hens have not been investigated. Therefore, this study aimed to analyze the mitigating effect of IA on intestinal damage caused by PFOA exposure and investigate the potential mechanisms of action.

MDA, the final product of lipid peroxidation, is the predominant marker indicating oxidative stress, and its concentration reflects the extent of oxidative stress-induced damage in the body [30]. In the present study, IA supplementation significantly reduced the PFOA-induced increase in MDA in serum and intestine, suggesting a protective effect of IA on the organism and intestinal tissues against oxidative damage. This effect may be due to changes in antioxidant enzyme activities. Studies have shown that PFOA reduces the activity of antioxidant enzymes such as GSH-Px and CAT, resulting in the inability of the body’s antioxidant capacity to load the production of free radicals [31]. This accelerates the generation of MDA, leading to lipid peroxidation damage. However, in this study, IA supplementation increased the activities of antioxidant enzymes such as GSH-Px, SOD and CAT. This suggests that IA may function as a potential antioxidant by enhancing antioxidant enzyme activities, thereby reducing MDA production and alleviating oxidative stress damage induced by PFOA in laying hens.

Increased oxidative stress compromises the structural integrity of the gut and disrupts gut barrier function. In this study, HE results demonstrated that PFOA exposure disrupted the intestinal structure in laying hens, as evidenced by villous shortening, slight crypt hyperplasia, and inflammatory cell infiltration. Similarly, previous studies have indicated that PFOA exposure induces significant toxic effects on the mouse colon, leading to substantial disruption of the intestinal tract’s structural integrity [32]. Notably, IA supplementation partially restored damage to the intestinal villi and crypt structures. Protection of the intestinal epithelium to maintain normal morphology and structure also necessitates an intact intestinal mucosal barrier. Mucins, particularly MUC2 secreted by goblet cells, are crucial components of the intestinal mucus barrier, shielding intestinal cells from harmful substances [33]. In this study, IA treatment significantly increased the reduction of jejunal goblet cells induced by PFOA, promoting mucus secretion. Fluorescence immunoassays and mRNA expression levels of MUC-2 further confirmed this. These results suggest that IA enhances the mucus barrier by restoring goblet cell count and upregulating the expression of MUC-2. Restoration of the mucus barrier effectively defends against external threats that disrupt the tight junctions between enterocytes. Tight junctions serve as the primary connection between intestinal epithelial cells and are crucial for regulating intestinal permeability and barrier function [34]. Intestinal TEM results showed that IA supplementation alleviated PFOA-induced impairment of tight junctions and microvilli compared to the PFOA group. The reduction of intestinal permeability indicators such as DAO and LPS also indicated the restorative effect of IA on intestinal barrier function. Furthermore, PFOA exposure reduced the expression of key tight junction proteins including *Claudin-1*, *Occludin* and *ZO-1* in the jejunum, whereas IA supplementation significantly reversed mRNA expression and restored intestinal barrier function. In conclusion, our findings indicate that IA alleviated the oxidative stress damage caused by PFOA to the intestine and restored intestinal barrier function and structural integrity.

A large body of evidence suggests that Nrf2-mediated oxidative stress and inflammatory pathways play a crucial role in maintaining gut functional integrity by reducing intestinal barrier damage [35,36]. To further explore whether the mitigating effect of IA on PFOA-induced intestinal injury is related to oxidative and inflammation-related pathways, we assessed the expression of Keap1/Nrf2/HO-1 oxidative pathway and related inflammatory factors. When stimulated by oxidative stress, Nrf2, which was originally bound to Keap1 in the cytoplasm, dissociates and translocates to the nucleus, regulating a series of antioxidant genes, including HO-1 and NQO1, and thus enhancing the organism’s antioxidant capacity [37,38]. The Keap1/Nrf2/HO-1 pathway is pivotal in modulating oxidative stress. PFOA has been reported to exert its reproductive toxicity by inhibiting the function of antioxidant enzymes through the downregulation of *Nrf2* expression in the mouse testis [39]. Our results showed that IA treatment significantly upregulated the *Nrf2* and *HO-1* expression and decreased *Keap1* levels compared to the PFOA group. In addition, the Keap1/Nrf2/HO-1 pathway is believed to directly reduce inflammation by inhibiting NF-κB activation and IL-1β production [40,41]. Consistent with this, we observed an increase in the expression of inflammatory markers such as *IL-1β* and *NF-κB* following PFOA treatment, which were significantly downregulated following IA treatment. Thus, our findings indicate that IA may play a crucial protective role in the intestine during PFOA exposure by alleviating oxidative stress and inflammatory responses through the Keap1/Nrf2/HO-1 pathway.

The gut microbiota is an important contributor to maintaining normal gut function and intestinal homeostasis [42]. Previous studies have demonstrated that exposure to PFOA disrupts the gut microbiota [27]. In this study, IA mitigated PFOA-induced dysregulation of the microbial ecology. PFOA induced a reduction in gut microbial α-diversity in this study, which was effectively restored by IA supplementation. Microbial diversity is a key factor in stabilizing the functioning of the intestinal microbiota, and significant changes in its values imply alterations in microbiota homeostasis and resilience [43]. The MDI index plot also further demonstrated that IA restored the gut flora disruption caused by PFOA. Additionally, β-diversity analysis revealed significant clustering among the treatment groups, suggesting that IA supplementation restored the structural composition of gut microflora disrupted by PFOA. Our results found that exposure to PFOA resulted in increased abundance of Firmicutes and decreased abundance of Bacteroides. Elevated Firmicutes to Bacteroides ratio (F/B) have been associated with intestinal flora dysbiosis and obesity [44]. Similar to this study, previous studies have found that exposure to pollutants such as PFAS leads to higher F/B ratios and is potentially associated with insulin resistance and obesity [45]. In fact, the dominant intestinal microbial communities in healthy laying hens are Bacteroidota and Firmicutes, whereas the large proliferation of Proteobacteria indicates a dysregulation of the microbiota ecology [46,47]. Importantly, in this study, IA significantly suppressed the increase in the abundance of Proteobacteria caused by PFOA exposure. Furthermore, recent findings indicate that IA modulates the gut microbiota by significantly reducing the abundance of pathogenic bacteria such as *Escherichia-Shigella* in the broiler gut [48]. Consistently, our results indicated that IA inhibited the proliferation of *Escherichia-Shigella*, which may be one of the reasons why IA alleviated gut microbiota disorders. Likewise, *Clostridium innocuum* [49] and *Ruminococcus torques* [50] are known to be associated with intestinal inflammation or infection, potentially pathogenic organisms whose abundance increases following PFOA exposure but decreases markedly following IA treatment. Moreover, prior research has demonstrated that subchronic exposure to PFOA reduces the abundance of beneficial bacteria, including *Lactobacillus*, in the intestines of mice. Similarly, this study revealed that IA partially restored the decrease in *Lactobacillus* caused by PFOA. These findings underscore IA’s role in regulating and optimizing microbial structure.

Correlation analyses were conducted to further investigate the relationship between gut microbiota and PFOA-induced intestinal oxidative stress and inflammation. Numerous studies have shown that the reduction of *Escherichia-Shigella* is closely linked to the alleviation of oxidative stress and a decrease in pro-inflammatory factors [51,52,53]. Similarly, in the present study, *Escherichia-Shigella* abundance was negatively correlated with Keap1/Nrf2/HO-1 pathway-related genes (*Nrf2* and *HO-1*) and positively correlated with intestinal inflammatory factors (*NF-κB*, *IL-1β*, *TNF-α*, and *IL-6*). We also observed that the increase in Proteobacteria abundance was accompanied by the downregulation of antioxidant genes (*HO-1* and *GPX1*) and the upregulation of pro-inflammatory factors (*TNF-α* and *NF-κB*). In conclusion, the correlation results showed significant associations between gut microbes, inflammatory factors, and Keap1/Nrf2/HO-1 pathway expression, suggesting that the protective role of IA against PFOA-induced oxidative stress and intestinal damage may be attributed to its modulation and optimization of microbial flora, particularly the inhibition of pathogenic bacterial proliferation.

## 4. Materials and Methods

### 4.1. Animals and Diets

The Animal Care and Use Committee of Zhejiang University (Approval No. ZJU20220310) approved all of the animal experiments conducted in this study. In total, 360 36-week-old Jingbai laying hens from the same batch were randomly divided into 4 groups: basal diet (CON), basal diet supplemented with 250 mg/kg PFOA (PF), basal diet supplemented with 5 g/kg IA (IA), and basal diet supplemented with 250 mg/kg PFOA and 5 g/kg IA (PF + IA). Each group comprised 6 replicates, with 15 hens per replicate. During the experimental period, hens were accommodated in three-tier cages measuring 45 × 45 × 45 cm, with three hens per cage. The ambient temperature was controlled at 24 ± 3 °C, with relative humidity maintained at 50–60%, and the lighting conditions were programmed for 16 h of light and 8 h of darkness per cycle. All hens received water and diet ad libitum. After 2 weeks of normal feeding, the testing period began and lasted for 8 weeks. The basal diet (Table S1) was formulated to meet or exceed the criteria established by the National Research Council (NRC, 1994).

### 4.2. Sample Collection

After the trial, six hens were randomly selected from each group, one from each replicate. Blood samples were collected from the wing vein, and serum was separated by centrifugation at 3500× *g* for 10 min. After euthanizing the hens, the intestines were isolated. The jejunum was fixed in paraformaldehyde and glutaraldehyde, and the mucosa was scraped for subsequent experimental analysis. The contents of the cecum were preserved in sterile tubes for microbiota analysis.

### 4.3. Assay of Antioxidant Indices

After preparing the jejunal mucosa into tissue homogenates, T-SOD (A001-3-2), GSH-Px (A005-1-2), and CAT activities (A007-1-1), as well as MDA content (A003-1-2) in the homogenate supernatant, were assessed using commercial kits (Jiancheng Bioengineering Institute, Nanjing, China).

### 4.4. Histological Observation

Jejunal tissues fixed in paraformaldehyde were paraffin-embedded and stained with hematoxylin and eosin as described previously [54]. Three typical fields with intact and straight villi were selected per section for analysis of crypt hyperplasia and villous atrophy. Specifically, villus height and crypt depth were assessed using light microscopy and ImageJ (1.53) software, and the ratio of villus height/crypt depth (VCR) was calculated. PAS staining was conducted to examine the goblet cells. Paraffin sections were deparaffinized, oxidized with periodic acid, stained with Schiff’s reagent for 25 min, and counterstained with hematoxylin before sealing the slides for microscopic examination. Jejunal tissues fixed in glutaraldehyde were prepared for transmission electron microscopy as described previously [55]. Briefly, after cutting, dehydration, infiltration, sectioning, and staining, images were captured using transmission electron microscope (JEOL, Tokyo, Japan).

### 4.5. Immunofluorescence Analyses

Paraffin sections of jejunum were deparaffinized and placed in Citric Acid Antigen Repair Solution (HKI0001, Hock Biotechnology Co., Ltd., Hangzhou, China) for antigen repair before being closed with 3% BSA (HKW2084, Hock Biotechnology Co., Ltd., Hangzhou, China) for 1 h at room temperature. Occludin antibody (dilution 1:500, 27260-1-AP, Proteintech), MUC-2 antibody (dilution 1:500, gtx100664, Genetex, Irvine, CA, USA), and Nrf2 antibody (dilution 1:500, 16396-1-AP, Proteintech, Sankt Leon-Rot, Germany) were then placed at 4 °C overnight. Subsequently, secondary Antibody (HKI0029, Hock Biotechnology Co., Ltd., Hangzhou, China) was applied for 2 h at room temperature and the nuclei were visualized by incubation with DAPI staining solution for 10 min in the dark. The sections were observed and photographed under a fluorescence microscope (NIKON, Shanghai, China).

### 4.6. ELISA Analysis

Serum DAO and LPS levels were measured using a chicken Lipopolysaccharide ELISA kit (ABclonal, Wuhan, China). Using the double-antibody sandwich method, the absorbance at 450 nm was assessed with an enzyme marker. Sample concentrations were calculated based on the standard curve.

### 4.7. RT-qPCR Analysis

Total RNA was extracted using the Trizol reagent, followed by cDNA synthesis using the cDNA Synthesis Kit (R223, Vazyme, Nanjing, China). RT-qPCR was conducted using a commercial kit (Q712, Vazyme, Nanjing, China) on a qRT-PCR instrument (Bio-Rad, Hercules, CA, USA). The data were analyzed using the 2^−ΔΔCT^ method, with primer sequences detailed in Table S2.

### 4.8. Analysis of Gut Microbiota

The microbiological analysis was conducted as previously described [55]. Briefly, DNA was extracted from cecal content samples and sequenced on the Illumina MiSeq PE300 platform (Illumina, San Diego, CA, USA). PLS-DA was used to analyze similarities among different groups and to investigate the significance of the differences between them. LEfSe analysis identified significantly abundant bacterial taxa among the groups (LDA score > 4, *p* < 0.05).

### 4.9. Statistical Analysis

Statistical analysis was conducted using SPSS 26.0, employing one-way ANOVA to test for multiple comparisons and Student’s *t*-test for comparisons between two groups, with significance set at *p* < 0.05. Values were presented as mean ± SEM. Image were generated using GraphPad Prism 9.5 software.

## 5. Conclusions

**Figure 11:**
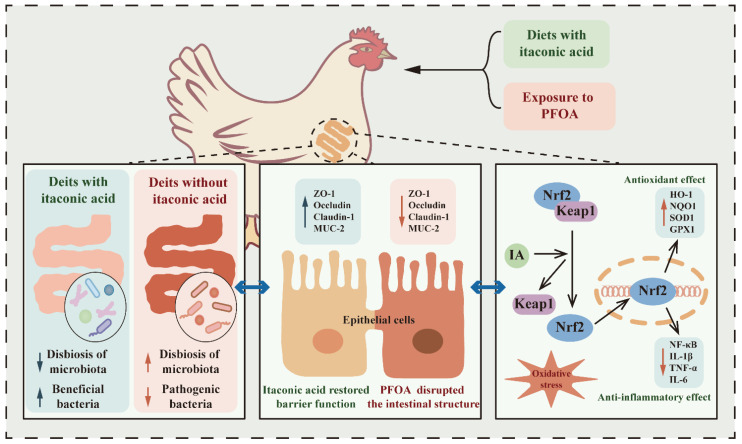
Schematic representation of potential mechanisms by itaconic acid alleviates intestinal damage and oxidative stress induced by PFOA.

In summary, this study illustrates that dietary IA supplementation could alleviate intestinal oxidative stress and inflammatory damage induced by PFOA exposure and restore intestinal barrier function and structural integrity. The protective effect of IA on the intestinal homeostasis may be attributed to the modulation of Keap1/Nrf2/HO-1 pathway via the gut microbiota and the inhibition of oxidative stress and inflammatory responses. These findings may provide new insights into IA as a novel antioxidant for improving intestinal health in laying hens (Figure 11).

## Data Availability

Data will be provided on reasonable request.

## Supplementary Materials

The following supporting information can be downloaded at: https://www.mdpi.com/article/10.3390/ijms25189826/s1.
