# Supplementary material for: Itaconic Acid Alleviates Perfluorooctanoic Acid-Induced Oxidative Stress and Intestinal Damage by Regulating the Keap1/Nrf2/Ho-1 Pathway and Reshaping the Gut Microbiota
Source: Int J Mol Sci. 2024 Sep 11;25(18):9826. doi: 10.3390/ijms25189826 (PMC11432532; doi:10.3390/ijms25189826)
Supplement: Supplementary file 1 [file ijms-25-09826-s001.zip › ijms-3143768-supplementary.pdf]

Table S1. Ingredients and nutrient level of the basal diet (air-dry basis, %)

| Item | Content |
| --- | --- |
| **Ingredient** | |
| Corn | 57.00 |
| Soybean meal | 24.00 |
| Wheat middling | 5.0 |
| Emulsified fat powder | 1.50 |
| Limestone | 9.00 |
| Dicalcium phosphate | 1.00 |
| Salt | 0.30 |
| DL-methionine | 0.20 |
| Premix [1] | 2.00 |
| Total | 100.00 |
| **Measured nutrients levels [2]** | |
| Metabolizable energy, Mcal/kg | 2.63 |
| Crude protein, % | 16.35 |
| Lysine, % | 0.83 |
| Methionine, % | 0.48 |
| Cysteine + methionine, % | 0.75 |
| Calcium, % | 3.61 |
| Total phosphorus, % | 0.67 |

[1] The premix provided the following per kg of the diet: vitamin A, 12500 IU; vitamin $D_3$, 4000 IU; vitamin $K_3$, 2 mg; thiamine, 1 mg; riboflavin, 8.5 mg; calcium pantothenate, 50 mg; niacin acid, 32.5 mg; pyridoxine, 8 mg; folic acid, 5 mg; $B_{12}$, 5 mg; choline chloride, 500 mg; iron, 60 mg; copper, 10 mg; manganese, 80 mg; zinc 80 mg; iodine 0.3 mg; and selenium 0.30 mg.

[2] Metabolizable energy (ME) in the nutrient levels were calculated values, while the others were measured values.

Table S2. Primers used for quantitative real-time PCR

| Gene | Primer Sequence (5' to 3') | Accession No. |
|---|---|---|
| *IL-1β* | F: TGCCTGCAGAAGAAGCCTCG<br>R: CTCCGCAGCAGTTTGGTCAT | NM_204524.1 |
| *IL-6* | F: GCAGGACGAGATGTGCAAGA<br>R: ACCTTGGGCAGGTTGAGGTT | NM_204628.1 |
| *IL-8* | F: AGCTGCTCTGTCGCAAGGTA<br>R: GCTTGGCGTCAGCTTCACATC | NM_205498.1 |
| *TNF-α* | F: CCCTACCCTGTCCCACAACC<br>R: TGGGCGGTCATAGAACAGCA | XM_046927265.1 |
| *NF-κB* | F: TGGAGAAGGCTATGCAGCTT<br>R: CATCCTGGACAGCAGTGAGA | NM_205134.1 |
| *IFN-γ* | F: CTCGCAACCTTCACCTCACCATC<br>R: CAGGAACCAGGCACGAGCTTG | NM_205149.1 |
| *ZO-1* | F: TATGAAGATCGTGCGCCTCC<br>R: GAGGTCTGCCATCGTAGCTC | XM_015278981.1 |
| *Claudin-1* | F: AAGTGCATGGAGGATGACCA<br>R: GCCACTCTGTTGCCATACCA | NM_001013611.2 |
| *Occludin* | F: TCATCGCCTCCATCGTCTAC<br>R: TCTTACTGCGCGTCTTCTGG | NM_205128.1 |
| *MUC-2* | F: AGCGAGATGT TGGCGATGAT<br>R: AAGTTGCCACACAGACCACA | NM_001318434.1 |
| *NRF2* | F: GGGACGGTGACACAGGAACAAC<br>R: GCTCTCCACAGCGGGAAATCAG | NM_205117.1 |
| *Keap1* | F: CATCGGCATCGCCAACTT<br>R: TGAAGAACTCCTCCTGCTTGGA | XM_025145847.1 |
| *HO-1* | F: GCTGGGAAGGAGAGTGAGAGGAC<br>R: GCGACTGTGGTGGCGATGAAG | NM_205344.1 |
| *NQO1* | F: TCGCCGAGCAGAAGAAGATTGAAG | NM_001277619.1 |

| | | |
|---|---|---|
| | R: CGGTGGTGAGTGACAGCATGG | |
| *GPX-1* | F: GCTGTTCGCCTTCCTGAGAG<br>R: GTTCCAGGAGACGTCGTTGC | NM_001277853.2 |
| *SOD-1* | F: TCTTACCGGACCACACTGCATC<br>R: ACGAGGTCCAGCATTTCCAGTTA | NM_205064.1 |
| *β-actin* | F: ACCGGACTGTTACCAACACC<br>R: CCTGAGTCAAGCGCCAAAAG | NM_205518.1 |
